# Spine Metastasis

**DOI:** 10.1155/2011/375097

**Published:** 2011-12-11

**Authors:** Alessandro Gasbarrini, Rudolf Beisse, Charles Fisher, Laurence Rhines

**Affiliations:** ^1^Depatment of Oncologic and Degenerative Spine Surgery, Rizzoli Orthopaedic Institute, 40136 Bologna, Italy; ^2^Spine Center Munich, Orthopedic Hospital München-Harlaching, Grünwalderstr aße 51, 81547, Munich, Germany; ^3^Division of Orthopaedic Spine Surgery, Department of Orthopaedics, The University of British Columbia, Vancouver, BC, Canada V6T 1Z4; ^4^Department of Neurosurgery, The University of Texas MD Anderson Cancer Center, Houston, TX 77030, USA

Up to 70% of patients with cancer will develop spine metastasis. Clinical presentations vary, but pain, instability, and neurologic deficit alone or in combination are usually manifested. General management options include analgesia or more comprehensive palliative care pathways, hormonal or chemotherapy, radiation therapy, and surgery. Metastatic patients are unique compared to patients in other domains of health care. For the most part, these patients cannot be cured and are on a palliative trail of uncertain duration and quality of life. Decisions around care in this patient population must be shared with the patient, loved ones, and a multidisciplinary team knowledgeable in the spectrum of interventions available and the evidence on which they are founded.

Because of the multitude of issues involved in these patients' treatment decision making is difficult and controversial and must be individualized. Several scoring systems or classifications have been developed over the past 2 decades to help guide physicians in making the right treatment choices for their patients. Although no one classification is comprehensive enough or has gone through exhaustive psychometric analysis, they do help guide physicians in determining some treatment options. Often they are based on life expectancy, general health or imaging parameters and not on the primary clinical outcome of interest—health-related quality of life (HRQOL). Although HRQOL has broad and varying definitions depending on what aspect you are focussing on, treatment of patients with spine metastases should be directed to improving generic HRQOL or a specific aspect of it, such as pain. Recently there has been a growth in HRQOL research in patients with spine metastases and this has helped direct treatment.

Another area of rapid growth has been in technology in both the radiation and surgical domains. Stereotactic radiosurgery, percutaneous vertebral augmentation, and minimally invasive surgery have added to the physician and surgeon's armamentarium. Where they stand in comparison to more conventional forms of treatment has not been clearly determined, but their impact on HRQOL has certainly been positive. The real challenge now lies in the development of a new paradigm in the management of spine metastases as new technology has expanded indications and provided potentially more options to improve HRQOL. 

In this special issue, we have invited seven papers that provide the most up-to-date and comprehensive information about the management of patients with spine metastases. Essential background has been provided by G. Maccauro and colleagues with a detailed and clear paper on physiopathology of spine metastasis, underlining the aspects related to epidemiology, pathogenesis, and prognosis. An exhaustive reference list guides the reader to a deeper knowledge on the issue.

L. M. Shah and K. L. Salzman have described the state of the art of imaging in spinal metastatic disease, underlining the role of new technology and innovation through CT, MRI and nuclear medicine such as FDG-PET/CT. Imaging actually plays a fundamental role in not only diagnosis but also treatment planning and is part of the multidisciplinary approach to the issue.

Metastatic tumors of the spine can be either intradural or extradural. Intramedullary spinal cord metastases are even rarer than bone spine malignancies. Optimal management is difficult to identify due to the variety of clinical situations and the lack of controlled studies. O. Kalita and colleagues show a review of the literature on this topic.

W. A. Hall and colleagues wrote an evidence-based review on stereotactic body radiosurgery that is emerging as an effective and safe treatment modality for spinal tumors, both primary and metastatic. C. A. Molina, P. S. Rose and J. H. Schwab report about the minimally invasive spine surgery (MISS). The first of them has performed a systematic review of the actual role of the procedure in the setting of spine metastases management. P. S. Rose and colleagues describe the surgical techniques used and possible combination with other procedures to gain the best possible result. J. H. Schwab deals with outcome evaluation in patients affected by spine metastases and treated with MISS. Good preliminary results reported are in favour of these techniques, but authors also underline the need for a multidisciplinary approach and a careful evaluation of the surgical indication.

In conclusion a global, contextualized, multidisciplinary approach to spinal metastases is essential if optimal HRQOL is to be achieved [1]. Furthermore, we must encourage and evaluate new technology so as to expand the options for this challenging and very deserving patient population. 

*Alessandro Gasbarrini* *Alessandro Gasbarrini* *Rudolf Beisse* *Rudolf Beisse* *Charles Fisher* *Charles Fisher* *Laurence Rhines* *Laurence Rhines*
